# Experimental Exploration of Multilevel Human Pain Assessment Using Blood Volume Pulse (BVP) Signals

**DOI:** 10.3390/s23083980

**Published:** 2023-04-14

**Authors:** Muhammad Umar Khan, Sumair Aziz, Niraj Hirachan, Calvin Joseph, Jasper Li, Raul Fernandez-Rojas

**Affiliations:** Human-Centred Technology Research Centre, Faculty of Science and Technology, University of Canberra, Canberra, ACT 2617, Australia; umar.khan@canberra.edu.au (M.U.K.); sumair.aziz@canberra.edu.au (S.A.); niraj.hirachan@canberra.edu.au (N.H.); calvin.joseph@canberra.edu.au (C.J.); u3242794@uni.canberra.edu.au (J.L.)

**Keywords:** pain classification, blood volume pulse (BVP), PPG, feature extraction, machine learning, pain intensity classification

## Abstract

Critically ill patients often lack cognitive or communicative functions, making it challenging to assess their pain levels using self-reporting mechanisms. There is an urgent need for an accurate system that can assess pain levels without relying on patient-reported information. Blood volume pulse (BVP) is a relatively unexplored physiological measure with the potential to assess pain levels. This study aims to develop an accurate pain intensity classification system based on BVP signals through comprehensive experimental analysis. Twenty-two healthy subjects participated in the study, in which we analyzed the classification performance of BVP signals for various pain intensities using time, frequency, and morphological features through fourteen different machine learning classifiers. Three experiments were conducted using leave-one-subject-out cross-validation to better examine the hidden signatures of BVP signals for pain level classification. The results of the experiments showed that BVP signals combined with machine learning can provide an objective and quantitative evaluation of pain levels in clinical settings. Specifically, no pain and high pain BVP signals were classified with 96.6% accuracy, 100% sensitivity, and 91.6% specificity using a combination of time, frequency, and morphological features with artificial neural networks (ANNs). The classification of no pain and low pain BVP signals yielded 83.3% accuracy using a combination of time and morphological features with the AdaBoost classifier. Finally, the multi-class experiment, which classified no pain, low pain, and high pain, achieved 69% overall accuracy using a combination of time and morphological features with ANN. In conclusion, the experimental results suggest that BVP signals combined with machine learning can offer an objective and reliable assessment of pain levels in clinical settings.

## 1. Introduction

Pain acts as a biomarker of numerous injuries, disorders, or stress conditions and assists the brain by providing a warning to operate against abnormalities in the body [1]. Pain serves as an autonomic warning mechanism to avoid likely alarming situations, for example, headaches that may indicate fatigue or stress and chest pains may be a sign of heart disease. Pain detection and sensation are essential functions of the human body and are based on the brain, spinal cord, and peripheral nervous system (PNS) [2]. Pain-processing mechanisms help prevent possible life-threatening conditions and are critical for survival [3]. These pain-processing mechanisms include recording and analyzing nociceptive sensory information, shifting the focus of attention toward pain processing, holding the information associated with pain in working memory, establishing quick communication with the motor system to prevent further harm, and avoiding future damage through memory encoding the current problem [4].

Pain is a highly subjective experience, and there is a lack of clinically available diagnostic tools to assess it objectively. Pain sensation and its tolerance to the body varies among individuals. There are two prominent methods employed for the assessment of pain in clinical procedures: self-reported and clinical assessment. In self-reported approaches, the pain of the patient is measured through verbal communication or numeric self-rating scales, such as verbal descriptor scales, the McGill pain questionnaire [5], or visual analog scales [6]. In contrast, pain measurement through clinical judgment relies on testing and learning from observations of the type, significance, and context of the patient’s pain perception [7]. The self-reporting method is considered the gold standard and presents the most valid assessment in clinical practices. However, clinical assessments can be used when self-reports are not available or are unreliable.

Self-report-based pain assessment furnishes significant clinical information and is considered valid in most cases, but it fails in certain scenarios [8]. For instance, patients who suffer from advanced dementia, communication disorders, intellectual disabilities, or are in a state of coma or in a minimally conscious condition may not be able to provide sufficient and accurate self-reports of pain [9,10,11]. An inaccurate assessment of these patients may lead to suboptimal or improper pain management, which may lead to other clinical complications, such as depression and psychological distress [12]. In addition, the self-reported pain assessment is highly subjective and very difficult to acquire continuously or in real time. Therefore, there is a need for more objective metrics to measure human pain in clinical practice to improve pain management, reduce risk factors, and contribute to a more valid and reliable diagnosis.

Previous studies have mainly focused on the detection and classification of pain through analysis of patterns of human behavior, such as facial expressions [13,14,15], body motions [16], and vocalizations [17]. Behavior-based assessment techniques are not suitable to be applied to patients suffering from paralysis or other motor diseases impacting their behaviors. Significant information can be extracted from observation of an individual’s face about the affective state, including pain. However, pain detection using facial expressions requires targeting a particular region of the subject’s face, which may become very complex and tedious in some clinical environments. In addition, facial expression-based pain assessment can be very difficult to use for subjects who are in a prone position; head posture and movement tracking are additional issues to consider. Keeping in view the aforementioned problems, research has also diverted toward developing physiological signal-based pain assessment approaches that do not depend on the individual’s behaviors [18,19,20].

Existing research includes pain analysis through various physiological signals. These physiological-based assessments includes electrodermal activity (EDA) [21], heart rate variability [22,23], electroencephalography (EEG) [24], electromyography (EMG) [25], and functional near-infrared spectroscopy fNIRS [4,7,26]. Most of the existing research employing physiological signals for pain assessment provides qualitative analysis for differentiating between pain and no pain conditions using machine learning [27]. In this context, machine learning has been fundamental for the success of the use of physiological signals in the study of pain; for a comprehensive review of machine learning in pain research, the interested reader is referred to [28]. The results of these physiological-based studies using machine learning demonstrate that the classification and identification of human pain are plausible.

Although current studies have shown promising results, there is still a lack of acceptable pain detection and classification method based on low-cost and convenient physiological signals. With the growing presence of wearable technology, it is possible to design a computer-aided diagnosis system for pain assessment using non-invasive wearable sensors. Photoplethysmography (PPG) is a commonly available sensing modality for physiological monitoring that provides blood volume pulse (BVP) information. As compared to impedance plethysmography, PPG does not need skin contact [29]. BVP captures even slight variations induced due to modulation perfusion of skin tissue using visible or IR sensing technology [30]. As compared to multi-channel systems (e.g., EEG, fNIRS), PPG is computationally efficient, as it has only one channel. Another advantage of PPG is that is less obstructive than other sensing technologies (e.g., ECG and EMG) in clinical settings, as it can be placed on the earlobe or the fingers.

Therefore, the objective of this study is to explore variations in pain intensities using BVP signals to develop an efficient pain classification system. The main contributions of this study are listed below:

1. A new BVP signal dataset featuring various pain intensities was recorded by inducing pain at different anatomical locations of healthy human subjects.
2. The exploration of time, frequency, and morphological features extracted from BVP as a potential indicator of various pain levels.
3. The comparison of a wide range of machine learning classification algorithms for recognition of pain in different experimental configurations.
4. The experimental analysis demonstrates that the BVP signal is a strong candidate to be employed for pain assessment in clinical settings.

This article is structured as follows: Section 2 “Materials and methods” provides details about the pain dataset and developed methodologies for the assessment of pain BVP signals. Section 3 “Results” illustrates the experimental results of the BVP signal analysis. The discussion of results and comparison with previous studies is also provided in Section 4. Finally, Section 5 concludes this article and provides future research directions.

## 2. Materials and Methods

### 2.1. Participants

Twenty-two participants (12 F/10 M) took part in the experiment. Their age ranged from 19 to 36 years old (mean age 27±4.19 std). No participants reported a prior history of neurological or psychiatric disorder, a current unstable medical condition, chronic pain, regularly taking medications, or being under medication at the time of testing. Participants were given a detailed explanation of the experimental procedures upon their arrival. Written informed consent was obtained before the start of the experiment. The experimental procedures involving human subjects described in this paper were approved by the University of Canberra’s Human Ethics Committee (number: 11837).

### 2.2. Experimental Procedure and Dataset

All experiments were conducted at the Human–Machine Interface Laboratory at the University of Canberra, Australia. The participants were seated on a chair in front of a table with both arms resting on the table. The blood volume pulse (BVP) sensor was placed on the middle finger of the left hand, the sensor was made by Biosignal plux (Lisbon, Portugal) [31]. On the right arm, the electrodes of a transcutaneous electrical nerve stimulation (TENS) machine (Medihightec Medical Co., Ltd., Taiwan) were placed on the inner forearm and on the back of the hand. These two anatomical locations will be used to explore the possibility to identify the source of pain in our future work. The location and intensity of pain stimulus were counterbalanced to avoid habituation to repeated experimental pain and to avoid confounding due to order effects.

**Figure 1:**
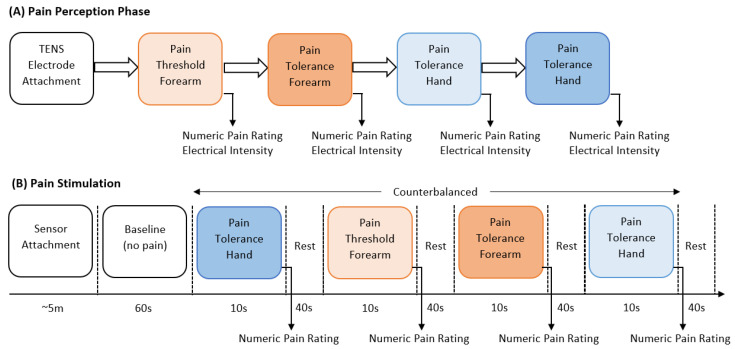
Schematic representation of stimulation and perception of pain.

The experiment consisted of two main parts, the identification of individual pain perceptions and the pain stimulation part. Figure 1 presents a schematic representation of the experimental procedure. In the first part of the experiment, pain perceptions were obtained using the quantitative sensory testing (QST) protocol [32]; no sensor was used in this part of the experiment. The QST protocol is used to determine an individual’s pain threshold and pain tolerance. We defined pain threshold (low pain) as the lowest stimulus intensity at which stimulation becomes painful, and pain tolerance (high pain) as the highest intensity of pain at which stimulus becomes unbearable [7]. The participants were exposed to gradually increasing stimulus and were instructed to verbally rate (0 = ‘no pain’, 10 = ‘maximum pain’) the pain intensity when the stimulation became painful (pain threshold) and then when the stimulation became unbearable (pain tolerance). The intensity of the TENS machine, in which the threshold and tolerance of pain occurred, was recorded to be used as the intensity during the stimulation part.

**Figure 2:**
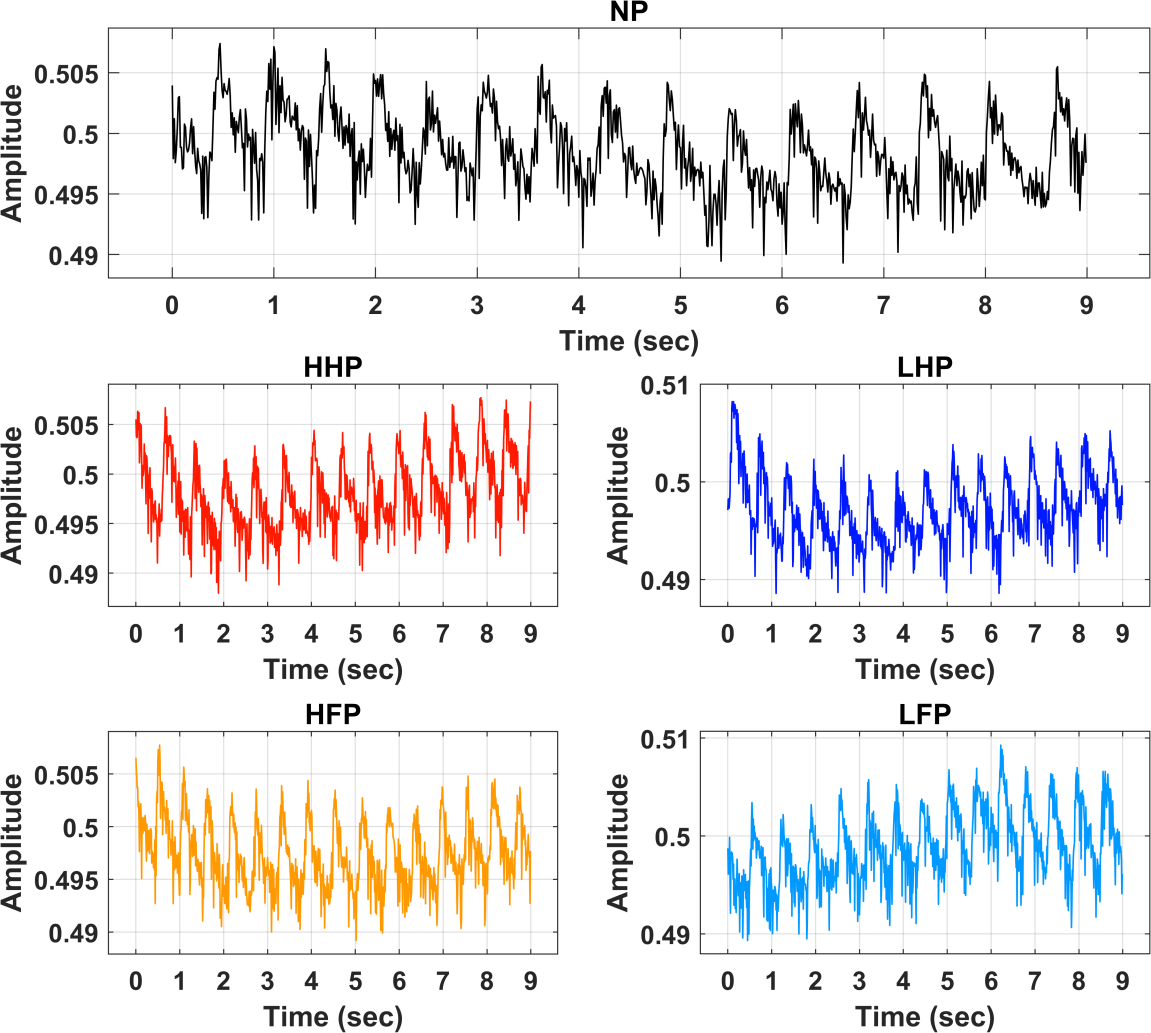
Raw BVP signals of No Pain (NP), High Hand Pain (HHP), High Forearm Pain (HFP), Low Hand Pain (LHP), and Low Forearm Pain (LFP).

In the second part, the pain intensity and anatomical location were studied. Before the start of the electrical pain stimulation sequence, the physiological sensor was placed on the hand of the participant. At the start of the experiment, a 60 s baseline period was recorded, in which the participants were instructed to remain calm; this baseline period served as the no-pain condition for the classification tasks. After that, the counterbalanced design alternated stimuli intensity (low or high) and location (forearm or hand). Six repetitions with a duration of 10 s for each stimulus were recorded. Immediately after each stimulus, the participants were asked to verbally rate the stimulation using the same scale (0 = no pain, 10 = maximum pain) used during the pain perception. After rating the pain intensity, a 40 s rest period was offered to allow all physiological signals to return to baseline. Each experiment lasted for approximately one hour (30 min preparation and individual pain perception, and 30 min pain stimulation experiment). Table 1 provides a summary with all details about the dataset employed in this study. Raw BVP signals of No Pain (NP), High Hand Pain (HHP), High Forearm Pain (HFP), Low Hand Pain (LHP), and Low Forearm Pain (LFP) are shown in Figure 2.

### 2.3. BVP Signal Preprocessing

**Figure 3:**
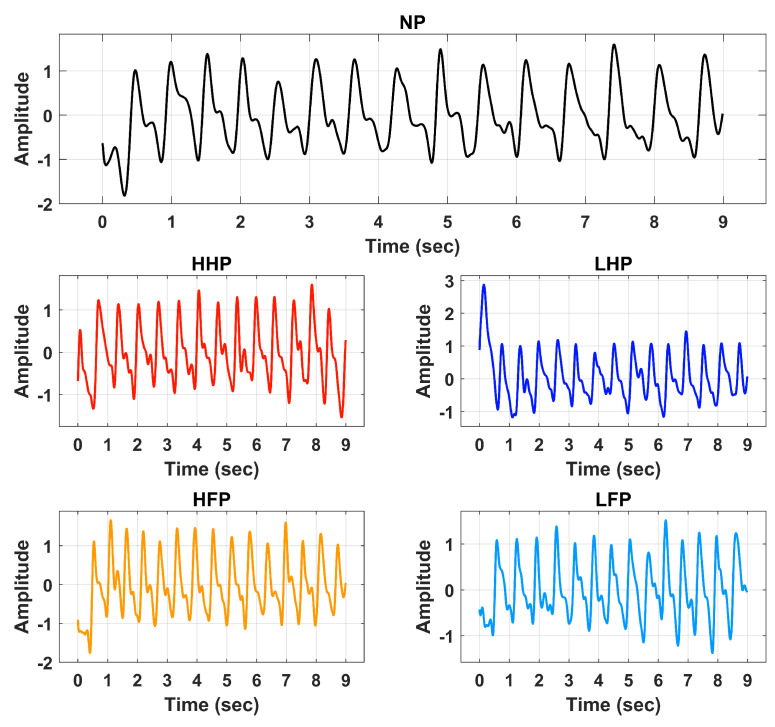
Processed signals of No Pain (NP), High Hand Pain (HHP), High Forearm Pain (HFP), Low Hand Pain (LHP), and Low Forearm Pain (LFP).

Signal preprocessing is a crucial step in signal analysis and classification frameworks. Preprocessing is performed to remove the unwanted frequencies and offsets incurred during data recording activities. In this research, the BVP signals of various pain levels were first preprocessed through an IIR band pass filter of the 4th order. The passband frequency range of 0.5 Hz to 5 Hz was selected for the filtration operation. The filter was designed to suppress the DC offset contained by the input signal. It also efficiently attenuates high-frequency components and harmonics. Figure 3 illustrates the preprocessed BVP signals of NP, HHP, HFP, LHP, and LFP.

### 2.4. Feature Extraction

Features express the most important patterns of the signal data in a binary, categorical, or continuous form. The objective of the feature extraction step is to represent signal data in a simplified and compact manner having maximum representation from the raw data. Feature extraction is performed after preprocessing and signal denoising operations. The extracted features are identified to possess minimum differences within the same class and maximum differences between centroids of other classes. In this study, we extracted features of various types from the BVP signal to better classify pain and its intensities. These features include time, frequency, and morphological type features (explained below). In general, pain causes sympathetic nervous system activation, leading to vasoconstriction and reduced blood flow to the affected area. This can result in a decrease in BVP amplitude in the affected area, indicating reduced blood volume. This variation in signal amplitude is better captured through time and morphological features. Frequency domain analysis of BVP signals can provide valuable insights into changes in the signal due to pain stimulation, particularly in terms of sympathetic and parasympathetic activity. For each classification task, the analysis is performed using an individual set of features as well as their different combination in order to determine the optimal features.

#### 2.4.1. Time Features

The shape of BVP is normally divided into two periods. The rising part of the wave is called the anacrotic phase, and the falling edge of the pulse is known as the catacrotic phase. The first part is related to the systole, and the second phase is concerned with diastole and wave reflections from the periphery. A set of twelve-time domain features such as mean (M), variance (V), skewness (S), kurtosis (K), crest factor (CF), shape factor (SF), impulse factor (IF), margin factor (MF), Shannon energy (SE), log energy (LE), mobility (Mob), and complexity (Comp) were extracted from BVP signal to represent the pain and non-pain signals in a compact manner [33]. Table 2 provides a mathematical description of all time-domain features used in this work.

#### 2.4.2. Frequency Features

Fourier transform maps the preprocessed BVP signal to the frequency domain and exposes the spectral information of the signal. This spectral information helps to extract discriminative features from the BVP signals about various pain conditions. In this study, we extracted a set of twelve frequency domain features, namely, spectral flux (SpF), spectral crest (SpC), spectral flatness (SpFt), spectral centroid (SpCent), spectral kurtosis (SpK), spectral spread (SpSp), spectral roll-off (SpR), spectral slope (SpS), spectral decrease (SpD), spectral entropy (SpE), and mean frequency (SpM) [34]. Mathematical formulation of all spectral features is provided in Table 2.

#### 2.4.3. Morphological Features

Morphological features represent the shape characteristics of the BVP signal of no pain and various pain categories. Analysis revealed that there exist significant differences in the dicrotic notch portion of the BVP signal for various cardiac conditions [35]. In this study, we extracted a group of twelve morphological features. Table 3 provides complete details of selected morphological features used in this study.

### 2.5. Classification Methods

Detection and classification of pain using extracted time, frequency, and morphological features were performed using shallow classifiers: support vector machines (SVM), adaboost (AB), random forest (RF), and fine-decision tree (Ftree), k-nearest neighbors (KNN), and different versions of artificial neural networks (ANNs). These algorithms are commonly employed for classification tasks in biomedical signals [36,37,38].

KNN is a type of supervised machine learning model typically used for regression and classification applications [33]. KNN determines the class of input data by computing the distance of current features will previous data points. The output class is predicted using class information of the close neighbors. KNN does not have any specific training algorithm, and all training data are used to compare the input features in the prediction stage; therefore, the computational cost of KNN is very high. The value of ‘K’ is selected to determine the number of neighbors to be considered for output prediction. In this work, we employed Fine-KNN (FKNN), where the value of K is set to 1, and Weighted-KNN (WKNN) uses a weighted distance metric to compute the similarity between data points.

Support vector machine (SVM) a supervised learning algorithm that adopts a hyperplane-based mechanism to classify features of various categories. A hyperplane is the form of a separation line designed to separate binary classes. SVM enhances the difference between classes using kernel trick. Data are translated/mapped to higher dimensions using non-linear kernel functions, such as quadratic, cubic, Gaussian, and radial basis functions. In this study, we used Linear-SVM (LSVM), Quadratic-SVM (QSVM), Cubic-SVM (CSVM), Gaussian-SVM (GSVM), and Radial-SVM (RSVM) [36,39,40].

Ensemble classification algorithms are based on numerous classifiers that train multiple hypotheses to address the same problem. RF is constructed using a set of decision tree classifiers to distribute the feature data into multiple classes fitting to tree branches [41]. Output prediction is based on the feature similarity of the random features. The AB (adaptive boosting) ensemble learning algorithm used for the classification of BVP signals of pain uses numerous iterations to construct a single composite powerful learner [42]. This model is created by adding a new weak learner in each round by adjusting the weighting vector in a way that the focus is on correctly predicting the observations that were misclassified in the previous round. As a result, an ensemble of various weak models is generated that has an overall better performance. AB is more resistant to the overfitting problem as compared to other machine learning classifiers.

Artificial neural networks (ANNs) are used to model the complex relationships between inputs and outputs or to find patterns in data [43,44]. Feed-forward neural networks consist of input, output, and hidden layers. The neurons in the input layer get features extracted from the BVP signal as input and pass them to fully connected hidden layers. Hidden layers fine-tune the input weightings until the neural network’s loss function is minimized. Information from the hidden layers is passed to the output layer, which depends on the number of classes in the given dataset. In this study, four types of neural network structures were used for the analysis of pain BVP signals. A narrow neural network (NN) consists of a single hidden layer of size 10. A wide neural network (WNN) is created using 100 neurons in a hidden layer. Two hidden layers, each with 100 neurons, were used in a bi-layered neural network (BLNN). A tri-layered neural network (TLNN) was constructed with three hidden layers, each having 100 neurons. All networks were trained using a backpropagation algorithm that iteratively minimized the mean square error between the output produced by feed-forward networks and target labels. Rectified linear unit (ReLU) was used as an activation function with an iteration limit set to 1000. In the present study, an experimental evaluation of BVP pain assessment was performed on 14 baseline ML models. The reason to maintain default parameters was to avoid building models that were highly optimized for our specific dataset. However, we report results for the five most prominent classification algorithms in all three learning tasks, i.e., QSVM, WKNN, AB, BLNN, and TLNN. The classification parameters of these models are shown in Table 4.

### 2.6. Leave One Subject out Cross-Validation

**Figure 4:**
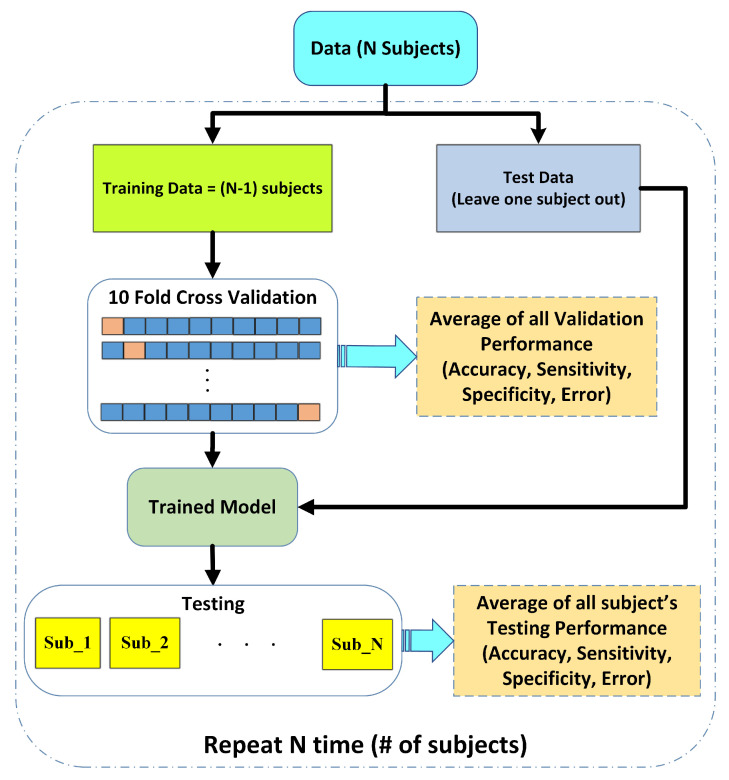
Performance evaluation scheme using leave one subject out cross validation (LOSOCV).

The performance of the proposed pain assessment system is evaluated using leave one subject out cross-validation (LOSOCV). Figure 4 illustrates the complete flow of the LOSOCV scheme and the details of the reported performance analysis in this article. In LOSOCV, features belonging to only one subject/person are employed as test sets (Ts), while the remaining subject data are combined to make a training set. LOSOCV provides reliable performance evaluation of the classification model by using the unseen subject’s data during the training stage. Mixing the same subject’s data in both test and training sets gives the classification model prior knowledge and results in biased high performance. Hence, LOSOCV provides a better-generalized assessment of the algorithms for unseen subject data. Validation is carried out using 10-fold cross validation (CV).

### 2.7. Performance Evaluation

Experiments were performed by considering pain BVP signals as a ‘positive class’ and ‘non-pain’ as a ‘negative class’. The overall accuracy (Acc) of the model is defined as follows:

(1) $$Accuracy=\frac{TP+TN}{TP+FP+TN+FN}$$

where True Positive (*TP*)—pain signals correctly predicted as pain; false positive (*FP*)—no-pain BVP signals misclassified as pain class; true negative (*TN*)—no-pain recordings are rightly identified as no-pain; and false negative (*FN*)—pain signals were incorrectly predicted as no-pain. Standard performance measures of sensitivity (Sen), specificity (Sp), positive predictive value (PPV), negative predictive value (NPV), and F1-score were also used to consolidate the experimental analysis.

### 2.8. Overview

**Figure 5:**
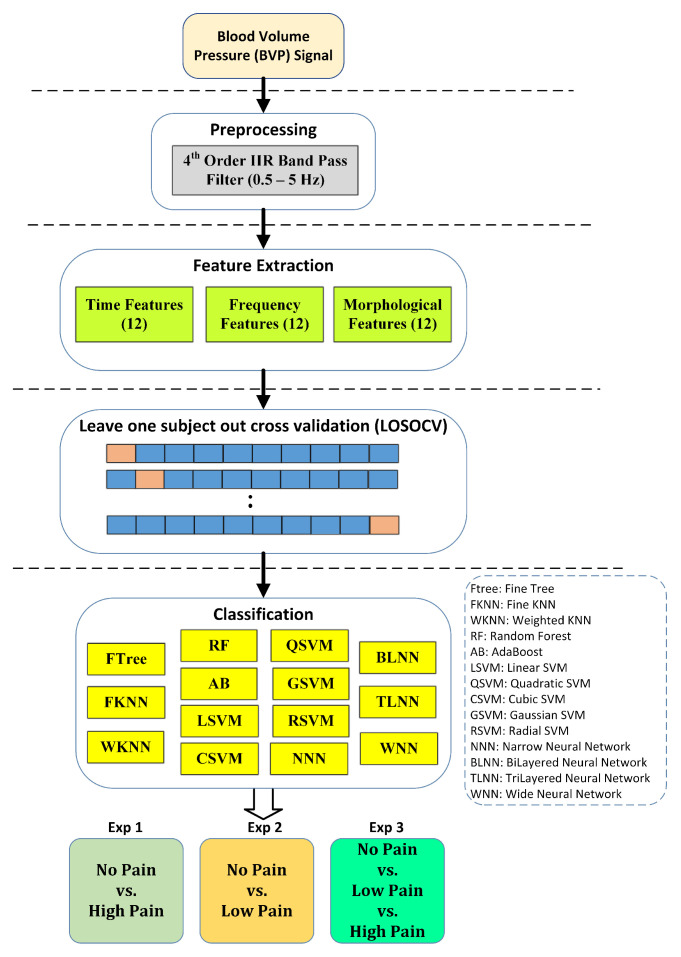
Design of study for assessment of pain using BVP signatures. Performance results are reported using the five most consistent classifiers.

Finally, Figure 5 illustrates the flowchart of the steps adopted in this work to develop a pain classification mechanism. In the first step, the blood volume pulse (BVP) signal is denoised using an IIR band pass filter with a cutoff range from 0.5 Hz to 5 Hz. Next, the denoised BVP signal is passed to the feature extraction stage, where a set of time, frequency, and morphological features are computed. The feature dataset was divided into training and testing segments using the leave one subject out cross-validation (LOSOCV) strategy. Individual feature sets and their various combinations were applied to train well-known classification methods, such as support vector machines, ensemble methods, neural networks, k-nearest neighbors, etc.

Three different experiments were performed for better assessment of BVP signals towards the identification of pain. Experiment 1 is a binary problem and aims to classify no pain against high pain. Experiment 2 aims to distinguish no pain vs. low pain classes of BVP signals. Experiment 3 is a multiclass problem and classifies no pain vs. low pain vs. high pain.

## 3. Results

Our motivation is to investigate the performance of time, frequency, and morphological features extracted from BVP features with well-known classification techniques. Comprehensive analysis is conducted in three experimental configurations. All experiments were performed in MATLAB 2020 running on a personal laptop (Intel Core i5 with 16 GB RAM). The experimental analysis from each experiment is discussed in detail in the following sections.

### 3.1. Experiment 1: No Pain vs. High Pain

**Figure 6:**
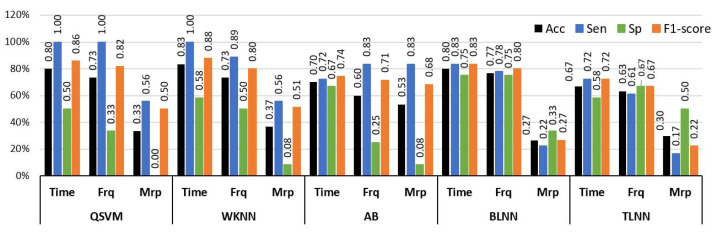
Performance evaluation with time, frequency (Frq), and morphological (Mrp) features using different classifiers for Experiment 1 (no pain vs. high pain). (QSVM: Quadratic-SVM, WKNN: Weighted-KNN, AB: AdaBoost, BLNN: bi-layered neural network, TLNN: tri-layered neural network).

The binary class experiment was designed to distinguish no pain (NP) and high pain (HP) BVP signals collected from arm and forearm positions. This makes the dataset of BVP signals more diverse and enables the algorithm to detect different signatures of pain originating from multiple locations. A set of time, frequency, and morphological features was tested separately with well-known classification methods. Figure 6 shows a graphical comparison of different classifiers with time, frequency, and morphological features. The best performance of 83.3% accuracy, 100% sensitivity, and 58.3% specificity was obtained via time domain features with the WKNN classifier. Frequency domain features offered 76.7% accuracy with BLNN and 73.3% when used in conjunction with WKNN and QSVM. Individual classification ability of morphological features was on the lower side as compared to time and frequency features.

The analysis of single-domain features exhibited that the use of these features independently was not adequate to provide discriminative information of pain from BVP signals (Figure 6). Therefore, the experimental analysis was extended by including feature sets that were composed of attributes from different domains. Table 5 provides a comprehensive performance evaluation of these combined features and classifiers for the classification of NP and HP BVP signals.

The highest performance results of 96.6% accuracy, 100% sensitivity, and 91.6% specificity were obtained through a hybrid feature set that consists of combined attributes computed from the time, frequency, and morphological domains and the TLNN classifier. The same feature set offered 83.3% mean accuracy with WKNN and 80% with BLNN classification methods (Table 5). A combination of time and frequency features showed 80% accuracy with both QSVM and WKNN classifiers. A combination of time and frequency features with QSVM and WKNN classifiers yielded 80% mean accuracy. An improvement in accuracy was observed by including morphological features with time and frequency features when employed with the TLNN classification model (from 73.3% to 96.6%).

**Figure 7:**
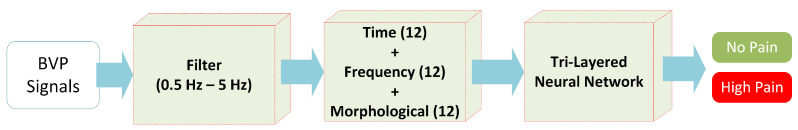
The proposed methodology for experiment 1 (no pain vs. high pain).

To summarize, Figure 7 shows the simplified view of the classification mechanism of HP and NP BVP signals through hybrid features (time+frequency+morphological) with the 3-layered neural network structure.

### 3.2. Experiment 2: No Pain vs. Low Pain

**Figure 8:**
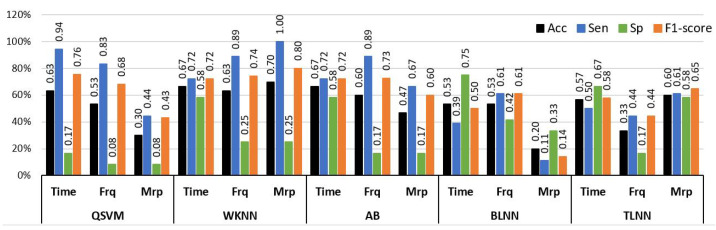
Performance evaluation with time, frequency (Frq), and morphological (Mrp) features using different classifiers for Experiment 2 (No Pain vs. Low Pain). (QSVM: Quadratic-SVM, WKNN: Weighted-KNN, AB: AdaBoost, BLNN: bi-layered neural network, TLNN: tri-layered neural network).

Experiment 2 was designed to analyze the difference between the no pain (NP) and low pain (LP) classes of BVP signals. The LP signals were recorded by stimulating in two sites, i.e., hand and forearm. This was done to increase the diversity of data recorded from the same subjects. This diversity also makes the classification tasks more challenging. Similarly to Experiment 1, a comprehensive analysis was performed using a set of time, frequency, and morphological features with a range of machine learning classifiers. The results of the analysis with individual feature sets are illustrated in Figure 8 in terms of accuracy, sensitivity, and specificity. The highest individual feature performance of 70% accuracy for distinguishing NP and LP BVP signals was obtained via morphological features and the WKNN classifier. The time and frequency domain features offered 66.6% and 63.3% accuracies with AB, and WKNN classifiers, respectively. It is important to highlight that only morphological features with WKNN achieved good accuracy of 70% and sensitivity value of 100%, but the specificity value dropped to 25%. However, this shows that morphological features have better recognition for positive class (sensitivity), which is LP in our case. A balanced performance of 72.2% sensitivity and 58% specificity was obtained with time features and AB classifier.

The individual feature analysis (Figure 8) demonstrated that a single type of feature was not ample for identifying hidden distinguishable content from BVP signals for NP and LP conditions. Again, the experimental analysis was extended by validating the classification algorithms through hybrid features that were constructed by combining features from different domains. Table 6 provides a comprehensive comparison of different classifiers with a combination of time, frequency, and morphological features. The highest results of 86.6% accuracy, 100% sensitivity, and 66% specificity were obtained through the AB ensemble classifier with a hybrid feature set consisting of time, frequency, and morphological features of BVP signals. While a fusion of time+morphological and frequency+morphological feature sets yielded, with QSVM classifiers, 83.3% and 80% accuracy, respectively. A combination of time+morphological features achieved a performance of 73.3% accuracy with the TLNN classifier.

**Figure 9:**
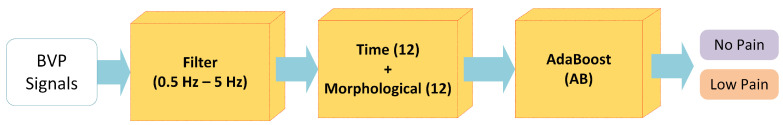
The proposed methodology for Experiment 2 (no pain vs. low pain).

The results obtained through the AB were the highest as compared to QSVM, WKNN, BLNN, and TLNN classifiers. Figure 9 summarizes the best-performing methodology for the classification of NP and LP BVP signals.

### 3.3. Experiment 3: No Pain vs. Low Pain vs. High Pain

This experiment was designed to identify different levels of pain (LP and HP) and baseline (NP) BVP signals. HP BVP dataset consists of High Hand Pain (HHP) and High Forearm Pain (HFP), and the LP class consists of BVP recordings of Low Hand Pain (LHP) and Low Forearm Pain (LFP). Again, the analysis of preprocessed BVP signals for these categories was performed by computing time, frequency, and morphological features and classifying them with a range of different machine-learning models.

**Figure 10:**
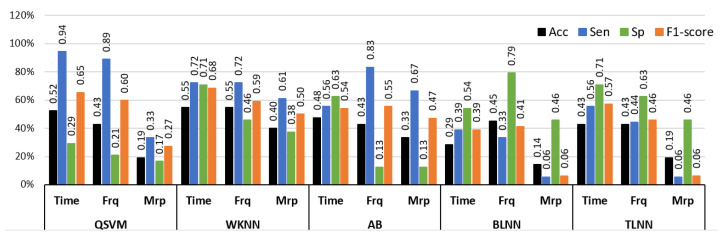
Performance evaluation with time, frequency (Frq), and morphological (Mrp) features using different classifiers for Experiment 3 (no pain vs. low pain vs. high pain). (QSVM: Quadratic-SVM, WKNN: Weighted-KNN, AB: AdaBoost, BLNN: bi-layered neural network, TLNN: tri-layered neural network).

Figure 10 illustrates the graphical comparison of individual feature sets with QSVM, WKNN, AB, BLNN, and TLNN classifiers. The highest accuracy of 54.7% was obtained via time features with the WKNN classifier. Time features with QSVM provided comparable results with 52.3% accuracy for identifying NP, LP, and HP BVP signals. The highest performance of frequency domain features was similar to the time features achieving 54.7% accuracy with the WKNN classifier. Individual feature analysis showed that the time and frequency domain features have better recognition capability as compared to morphological features.

In order to perform successful multiclass classification of NP, LP, and HP BVP signals, time, frequency, and morphological features were further examined by using hybrid features composed of multiple domains. Table 7 provides a comprehensive analysis of results for multilevel pain assessment using a combination of two or more feature sets with QSVM, WKNN, AB, BLNN, and TLNN classifiers. The overall best performance of 69% accuracy, 83.3% sensitivity, and 75% specificity were obtained using a combination of time and morphological features with a BLNN classifier. The combination of time+frequency+morphological features also provided comparable results of 64% accuracy with WKNN and 61% accuracy with TLNN.

**Figure 11:**
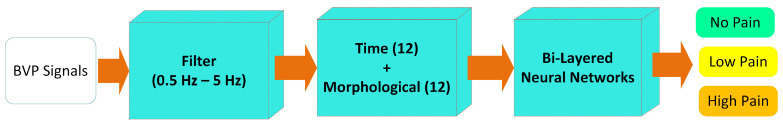
The proposed methodology for Experiment 3 (no pain vs. low pain vs. high pain).

Figure 11 illustrates the overall best-performing framework that consists of the filtration stage, a combination of time and morphological features and the BLNN classifier.

## 4. Discussion

In this study, we employed different experimental configurations for the assessment of pain intensities using BVP signals. BVP signals were analyzed with various time, frequency, and morphological features using a diverse range of classification methods. A summary of the best results for all experiments is provided in Table 8.

Experiment 1 (NP vs. HP) using BVP signals obtained the best results of 96.6% accuracy using a hybrid of time, frequency, and morphological features with the TLNN classifier. Direct comparisons with other studies are difficult because of the use of different experimental conditions (e.g., thermal, mechanical, or electrical stimuli), BVP acquisition systems, sampled populations with different demographics, validation methods, and classification models. However, in relation to other similar studies, our study presented comparable results. For example, Cao et al. [45] performed a classification of No Pain versus high-intensity pain using PPG signals and reported an accuracy of 79.4%. The authors employed empirical mode decomposition-based features combined with SVM for classification. Thiam et al. [46] reported 66% accuracy for the classification of No Pain (baseline) against high-intensity pain using respiratory signals. In comparison to [45,46], the results of our methods are significantly high, i.e., 96.6%. It is important to mention that 65 features were used in [46] as compared to our 36 features. Compared to the results provided in [45,46], our proposed framework achieved 17.2% and 36% increases, respectively.

Experiment 2 was designed to discriminate BVP signal features for NP and Low Pain (LP) states. The proposed method achieved 86.6% accuracy using a combination of time, frequency, and morphological features with the AdaBoost (AB) classifier. In comparison to published studies, Cao et al. reported 81.4% accuracy for no pain versus low-intensity pain using an SVM classifier [45]. In another study [46], authors obtained 50% accuracy for respiratory signal-based low-intensity pain assessment. The proposed method in this study for the binary classification of no pain and low pain levels using BVP signals provided better results.

Experiment 3 was designed to explore the discriminating power of BVP signals for multi-levels of pain intensities with baseline (no pain). The proposed method obtained 69% accuracy for a three-class problem (NP vs. LP vs. HP) using a combination of time and morphological features and a BLNN classifier. As compared to the first two binary experiments, the performance of the multi-class model is slightly low; however, this was expected, as the baseline accuracy in the three-class classification problem would be 33.33% or 1/3. Another explanation for the low accuracy might be due to the similarities between BVP signals of various pain intensity levels. To our best knowledge, the existing research lacks multi-class pain analysis. Most of the existing research followed a binary classification approach where baseline (no pain) was compared with various pain levels [45,47].

Previous research on pain assessment utilizing PPG signals were unable to introduce models capable of distinguishing between various pain signatures. This deficiency is a crucial concern for pain assessment within the human body, considering the various origins, intensities, and durations of pain (such as peripheral, visceral, emotional, and phantom pain) [4]. Each pain type exhibits unique signatures and is transmitted to the central nervous system through distinct sensory receptors. Therefore, developing machine learning models that can differentiate between multiple pain signatures with varying intensities would be more practical for real-life scenarios. This is particularly critical for patients who are unable to communicate verbally, such as those in a coma or with advanced dementia, or when the source of pain is unclear [48].

The selection and appropriate use of different features and machine learning models significantly contribute towards better performance. In our research, we found that the best-performing models for all experiments include time and morphological features that show their valuable contribution. Typically, pain triggers the activation of the sympathetic nervous system, which induces vasoconstriction and reduces blood flow to the area of pain. Consequently, there is a reduction in the amplitude of BVP in the affected region, reflecting a decrease in blood volume. The changes in signal amplitude are more accurately represented by examining the time and morphological features of the BVP signal. This is confirmed by the findings of this study as well.

Preliminary results presented in this study are promising; however, we acknowledge that this study presents some limitations that should be addressed in our future research. Confounding factors, such as stress or anxiety, can affect the quality of the data, in particular, due to anticipation of the upcoming pain stimulation. Therefore, in our future research experiments, we will try to minimize the participants’ stress as much as possible by asking subjects to keep their eyes closed to avoid pain anticipation [49]. Another limitation of the current study is the short length of pain stimulation (∼9 s) used in this experiment, in clinical contexts pain may have variable onset dynamics; thus, employing stimuli of different lengths will be more realistic in our future research. In the present study, baseline ML classification algorithms were used for the analysis; however, in future work, we will analyze the prediction performance by tuning the hyperparameters of classifiers. Finally, we will validate the obtained features to improve their feasibility in more realistic scenarios and their physiological significance and will include participants with a broader age range, so the learning model could generalize better to different populations.

## 5. Conclusions

In this study, we aimed to assess pain and its intensities using BVP signals in a scenario that is independent of the person being tested. To achieve this, we collected a new dataset that consisted of BVP recordings from healthy individuals who experienced various levels of pain induced in different locations of their bodies. Our findings demonstrate that BVP signatures exhibit good performance and offer a desirable trade-off between computational cost and accuracy, compared to other modalities. We conducted different experiments with varying pain configurations, utilizing a wide range of features and classifiers. The results of the experiments revealed classification performances of 96.6% (no pain vs. high pain), 86.66% (no pain vs. low pain), and 69% (no pain vs. low pain vs. high pain). These findings demonstrate the potential of BVP signals for pain assessment tasks.

In the future, our research group aims to explore more advanced feature descriptors using BVP and other modalities. We will also explore more advanced classification techniques (e.g., deep learning) that can help us improve the current results [50]. Our future work will also target pain assessment through the inclusion of non-communicable patients and newborns. We also plan to design a portable embedded system for the accurate classification of pain levels using wearables.

## Figures and Tables

**Table 1:** Summary of the dataset used for the detection and classification of pain.

Category	Details
Sampling rate	100 Hz
Number of subjects	22
Stimulation type	Electrical
Stimulation location	Hand and Forearm
Pain categories/classes	1. HP—High Pain (Pain tolerance or maximum level of Pain the subject can handle)
	2. LP—Low Pain (Pain threshold or minimum level of pain)
	3. NP—No Pain (baseline data without stimulation)
Signal type	Blood Volume pulse (BVP)
Length of each signal	9 s
Number of signals per class	NP: 396
	HP: 216
	LP: 216
Signals per subject	6 subjects have three signals per pain (HP/LP) class.
	14 subjects have six signals per pain (HP/LP) class.
	18 signals per subject for NP class.

**Table 2:** Mathematical description of time and spectral features used in this work.

Time Features	Spectral Features
$Mob=\sqrt{\frac{V\left(\frac{dP(t)}{dt}\right)}{V(P(t))}}$	$SpS=\frac{\sum_{k=c_{1}}^{c_{2}}\left(f_{q}-SpM\right)\left(F_{q}-\mu_{F}\right)}{\sum_{k=c_{1}}^{c_{2}}{\left(f_{q}-SpM\right)}^{2}}$
$M=\frac{1}{n}\sum_{i=1}^{n}P_{i}$	$SpD=\frac{\sum_{q=c_{1}+1}^{c2}\frac{F_{q}-F_{c_{1}}}{q-1}}{\sum_{q=c_{1}}^{c_{2}}F_{q}}$
$K=\frac{1}{n}\frac{\sum_{i=1}^{n}{\left(P_{i}-M\right)}^{4}}{\sigma^{4}}$	$SpF={\left(\sum_{q=c_{1}}^{c_{2}}|F_{q}\left(t\right)-F_{q}{\left(t-1\right)|}^{p}\right)}^{\frac{1}{p}}$
$CF=\frac{P_{peak}}{P_{rms}}$	$SpSk=\frac{\sum_{q=c_{1}}^{c_{2}}{\left(f_{q}-SpCent\right)}^{3}F_{q}}{\mu^{3}\sum_{q=c_{1}}^{c_{2}}F_{q}}$
$SF=\frac{P_{rms}}{P_{am}}$	$SpCent=\frac{\sum_{q=c_{1}}^{c_{2}}f_{q}.F_{q}}{\sum_{q=c_{1}}^{c_{2}}F_{q}}$
$IF=\frac{P_{peak}}{P_{am}}$	$SpK=\frac{\sum_{q=b_{1}}^{c_{2}}{\left(f_{q}-SpCent\right)}^{4}F_{q}}{\mu^{4}\sum_{q=c_{1}}^{c_{2}}F_{q}}$
$MF=\frac{P_{peak}}{{P_{am}}^{2}}$	$SpSp=\sqrt{\frac{\sum_{q=c_{1}}^{c_{2}}{\left(f_{q}-SpCent\right)}^{2}}{\sum_{q=c_{1}}^{c_{2}}F_{q}}}$
$LE=\sum_{i=1}^{n}log\left(P_{i}^{2}\right)$	$SpR=i\;s.t.\sum_{q=c_{1}}^{i}\left|F_{q}\right|=k\sum_{q=c_{1}}^{c_{2}}F_{q}$
$S=\frac{1}{n}\frac{\sum_{i=1}^{n}{\left(P_{i}-M\right)}^{3}}{\sigma^{3}}$	$SpC=\frac{max(F_{q\in [c_{1},c_{2}]})}{\frac{1}{c_{2}-c_{1}}\sum_{k=c_{1}}^{c_{2}}F_{q}}$
$V=\frac{\sum_{i=1}^{n}{\left(P_{i}-M\right)}^{2}}{n}$	$SpFt=\frac{{\left(\prod_{q=c_{1}}^{c_{2}}F_{q}\right)}^{\frac{1}{c_{2}-c_{1}}}}{\frac{1}{c_{2}-c_{1}}\sum_{q=c_{1}}^{c_{2}}F_{q}}$
$SE=-\sum_{i=1}^{n}P_{i}^{2}log\left(P_{i}^{2}\right)$	$SpM=\frac{\sum_{j=1}^{N}f_{j}F_{j}}{\sum_{j=1}^{N}F_{j}}$
$Comp=\frac{Mob(\frac{dP(t)}{dt})}{Mob(P(t))}$	$SpE=\frac{-\sum_{k=c_{1}}^{c_{2}}F_{k}log\left(s_{k}\right)}{log(c_{2}-c_{1})}$

*P_am_* is the absolute mean, *P_peak_* is the peak value, and *P_rms_* is the root mean square value of the preprocessed BVP signal *P*. *F_q_* and *f_q_* are the spectral and frequency values (Hz) at bin *q*, respectively. *μ_s_* is the mean spectral value, *p* is the norm type, and *c_1_* and *c_2_* are band edges.

**Table 3:** Details of morphological features extracted from BVP signals.

Abbreviation	Details
SDNN	The standard deviation of intervals
RMSSD	The square root of the mean of the squares of the successive differences between adjacent intervals
SDSD	The standard deviation of the successive differences between adjacent intervals
NN50	The number of pairs of successive intervals that differ by more than 50 ms
pNN50	The proportion of NN50 divided by the total number of intervals
NN20	The number of pairs of successive intervals that differ by more than 20 ms
pNN20	The proportion of NN20 divided by the total number of intervals
VLF	Total spectral power of all intervals between 0 and 0.04 Hz
LF	Total spectral power of all intervals between 0.04 and 0.15 Hz
HF	Total spectral power of all intervals between 0.15 and 0.4 Hz
SD1	The standard deviation of the Poincaré plot perpendicular to the line of identity
SD2	The standard deviation of the Poincaré plot along to the line of identity

**Table 4:** Hyperparameters used for each classifier.

Classifier	Parameters
QSVM	Kernel function: Polynomial, Polynomial order: 2, Box constraint: 1, Standardization: True, Coding: one vs. one
WKNN	Distance: Euclidean, Number of neighbors: 10, Distance weight: Sqauared inverse, Standardization: True
AB	Method: AdaBoost M1, Maximum number of splits: 20, Number of learning cycles: 30, Learning rate: 0.1
BLNN	Layer sizes: [100 100], Activation function: ReLU, Iteration limit: 1000, Standardization: True
TLNN	Layer sizes: [100 100 100], Activation function: ReLU, Iteration limit: 1000, Standardization: True

**Table 5:** Performance evaluation with combinations of time, frequency (Frq), and morphological (Mrp) features using different classifiers for Experiment 1 (no pain vs. high pain). (QSVM: Quadratic-SVM, WKNN: Weighted-KNN, AB: AdaBoost, BLNN: Bi-layered Neural Network, TLNN: Tri-layered Neural Network). Bold text indicates the best results.

Classifier	Feature Combination	Acc	Sen	Sp	PPV	NPV	F1-Score
QSVM	Time + Frq	80.00	100.00	50.00	75.00	100.00	85.71
	Time + Mrp	73.33	83.33	58.33	75.00	70.00	78.95
	Frq + Mrp	53.33	66.67	33.33	60.00	40.00	63.16
	Time + Frq + Mrp	70.00	88.89	41.67	69.57	71.43	78.05
WKNN	Time + Frq	80.00	88.89	66.67	80.00	80.00	84.21
	Time + Mrp	80.00	94.44	58.33	77.27	87.50	85.00
	Frq + Mrp	76.67	100.00	41.67	72.00	100.00	83.72
	Time + Frq + Mrp	83.33	100.00	58.33	78.26	100.00	87.80
AB	Time + Frq	66.67	66.67	66.67	75.00	57.14	70.59
	Time + Mrp	60.00	55.56	66.67	71.43	50.00	62.50
	Frq + Mrp	70.00	88.89	41.67	69.57	71.43	78.05
	Time + Frq + Mrp	63.33	55.56	75.00	76.92	52.94	64.52
BLNN	Time + Frq	76.67	83.33	66.67	78.95	72.73	81.08
	Time + Mrp	66.67	61.11	75.00	78.57	56.25	68.75
	Frq + Mrp	63.33	55.56	75.00	76.92	52.94	64.52
	Time + Frq + Mrp	80.00	83.33	75.00	83.33	75.00	83.33
**TLNN**	Time + Frq	73.33	66.67	83.33	85.71	62.50	75.00
	Time + Mrp	63.33	61.11	66.67	73.33	53.33	66.67
	Frq + Mrp	70.00	88.89	41.67	69.57	71.43	78.05
	**Time + Frq + Mrp**	**96.67**	**100.00**	**91.67**	**94.74**	**100.00**	**97.30**

**Table 6:** Performance evaluation with combinations of time, frequency (Frq), and morphological (Mrp) features using different classifiers for Experiment 2 (no pain vs. low pain). (QSVM: Quadratic-SVM, WKNN: Weighted-KNN, AB: AdaBoost, BLNN: bi-layered neural network, TLNN: tri-layered neural network). Bold text indicates the best results.

Classifier	Feature Combination	Acc	Sen	Sp	PPV	NPV	F1-Score
QSVM	Time + Frq	70.00	100.00	25.00	66.67	100.00	80.00
	Time + Mrp	83.33	100.00	58.33	78.26	100.00	87.80
	Frq + Mrp	80.00	100.00	50.00	75.00	100.00	85.71
	Time + Frq + Mrp	76.67	100.00	41.67	72.00	100.00	83.72
WKNN	Time + Frq	63.33	83.33	33.33	65.22	57.14	73.17
	Time + Mrp	73.33	94.44	41.67	70.83	83.33	80.95
	Frq + Mrp	70.00	100.00	25.00	66.67	100.00	80.00
	Time + Frq + Mrp	66.67	100.00	16.67	64.29	100.00	78.26
**AB**	Time + Frq	66.67	72.22	58.33	72.22	58.33	72.22
	Time + Mrp	80.00	83.33	75.00	83.33	75.00	83.33
	Frq + Mrp	73.33	100.00	33.33	69.23	100.00	81.82
	**Time + Frq + Mrp**	**86.67**	**100.00**	**66.67**	**81.82**	**100.00**	**90.00**
BLNN	Time + Frq	46.67	50.00	41.67	56.25	35.71	52.94
	Time + Mrp	63.33	83.33	33.33	65.22	57.14	73.17
	Frq + Mrp	63.33	61.11	66.67	73.33	53.33	66.67
	Time + Frq + Mrp	70.00	94.44	33.33	68.00	80.00	79.07
TLNN	Time + Frq	46.67	50.00	41.67	56.25	35.71	52.94
	Time + Mrp	73.33	88.89	50.00	72.73	75.00	80.00
	Frq + Mrp	63.33	72.22	50.00	68.42	54.55	70.27
	Time + Frq + Mrp	60.00	83.33	25.00	62.50	50.00	71.43

**Table 7:** Performance evaluation with combinations of time, frequency (Frq), and morphological (Mrp) features using different classifiers for Experiment 3 (No Pain vs. Low Pain vs. High Pain). (QSVM: Quadratic-SVM, WKNN: Weighted-KNN, AB: AdaBoost, BLNN: bi-layered neural network, TLNN: tri-layered neural network. Bold text indicates the best results.

Classifier	Feature Combination	Acc	Sen	Sp	PPV	NPV	F1-Score
QSVM	Time + Frq	54.76	88.89	50.00	57.14	85.71	69.57
	Time + Mrp	50.00	61.11	62.50	55.00	68.18	57.89
	Frq + Mrp	50.00	83.33	37.50	50.00	75.00	62.50
	Time + Frq + Mrp	52.38	83.33	45.83	53.57	78.57	65.22
WKNN	Time + Frq	52.38	61.11	66.67	57.89	69.57	59.46
	Time + Mrp	61.90	94.44	54.17	60.71	92.86	73.91
	Frq + Mrp	61.90	100.00	45.83	58.06	100.00	73.47
	Time + Frq + Mrp	64.29	94.44	58.33	62.96	93.33	75.56
AB	Time + Frq	35.71	55.56	54.17	47.62	61.90	51.28
	Time + Mrp	38.10	72.22	54.17	54.17	72.22	61.90
	Frq + Mrp	47.62	88.89	25.00	47.06	75.00	61.54
	Time + Frq + Mrp	40.48	66.67	58.33	54.55	70.00	60.00
**BLNN**	Time + Frq	40.48	50.00	75.00	60.00	66.67	54.55
	**Time + Mrp**	**69.05**	**83.33**	**75.00**	**71.43**	**85.71**	**76.92**
	Frq + Mrp	42.86	38.89	83.33	63.64	64.52	48.28
	Time + Frq + Mrp	57.14	88.89	66.67	66.67	88.89	76.19
TLNN	Time + Frq	54.76	61.11	87.50	78.57	75.00	68.75
	Time + Mrp	52.38	61.11	83.33	73.33	74.07	66.67
	Frq + Mrp	59.52	72.22	66.67	61.90	76.19	66.67
	Time + Frq + Mrp	61.90	77.78	70.83	66.67	80.95	71.79

**Table 8:** Summary of best-performing features and classification methods for all experiments. Experiment 1: no pain vs. high pain, Experiment 2: no pain vs. low pain, Experiment 3: no pain vs. low pain vs. high pain. Bold text indicates significant results.

Features	FVL	Experiment 1	Experiment 2	Experiment 3
Time	12	WKNN: 83.3%	WNN: 70%	WKNN: 54.7%
Frq	12	WNN: 76.6%	WKNN: 63.3%	NNN: 57.1%
Mrp	12	AB: 53.3%	LSVM: 63.3%	LSVM: 45.2%
Time + Frq	24	BLNN: 76.6%	QSVM: 70.0%	TLNN: 54.7%
Time + Mrp	24	QSVM:73.3%	QSVM: 83.3%	**BLNN: 69.04%**
Frq+ Mrp	24	FKNN: 83.33%	WNN: 73.33%	FKNN: 61.9%
Time + Frq + Mrp	36	**TLNN: 96.66%**	**AB: 86.66%**	WKNN: 64.28%

## Data Availability

The data that support the findings of this study are available from R.F.R., upon reasonable request.
